# Challenges of HIV diagnosis and management in the context of pre‐exposure prophylaxis (PrEP), post‐exposure prophylaxis (PEP), test and start and acute HIV infection: a scoping review

**DOI:** 10.1002/jia2.25419

**Published:** 2019-12-18

**Authors:** Tamara Elliott, Eduard J Sanders, Meg Doherty, Thumbi Ndung'u, Myron Cohen, Pragna Patel, Gus Cairns, Sarah E Rutstein, Jintanat Ananworanich, Colin Brown, Sarah Fidler

**Affiliations:** ^1^ Imperial College London London United Kingdom; ^2^ Imperial College Healthcare NHS Trust London United Kingdom; ^3^ Kenya Medical Research Institute‐Wellcome Trust Research Programme Kilifi Kenya; ^4^ Nuffield Department of Medicine University of Oxford Oxford United Kingdom; ^5^ Department of HIV and Global Hepatitis Programme WHO Geneva Switzerland; ^6^ Africa Health Research Institute Durban South Africa; ^7^ HIV Pathogenesis Programme Doris Duke Medical Research Institute University of KwaZulu‐Natal Durban South Africa; ^8^ The Ragon Institute of Massachusetts General Hospital Massachusetts Institute of Technology and Harvard University Cambridge MA USA; ^9^ Max Planck Institute for Infection Biology Berlin Germany; ^10^ Department of Internal Medicine Division of Infectious Diseases UNC School of Medicine University of North Carolina At Chapel Hill Chapel Hill NC USA; ^11^ Division of Global HIV and TB Centers for Disease Control and Prevention Atlanta GA USA; ^12^ NAM Aidsmap London United Kingdom; ^13^ PrEP in Europe Initiative London United Kingdom; ^14^ U.S. Military HIV Research Program Walter Reed Army Institute of Research Silver Spring MD USA; ^15^ Henry M. Jackson Foundation for the Advancement of Military Medicine Bethesda MD USA; ^16^ National Infection Service, Public Health England London United Kingdom; ^17^ Department of Infection Royal Free London NHS Foundation Trust London United Kingdom; ^18^ Imperial College NIHR BRC London United Kingdom

**Keywords:** Acute HIV infection, pre‐exposure prophylaxis, post‐exposure prophylaxis, immediate antiretroviral therapy, HIV testing algorithms, indeterminate HIV test

## Abstract

**Introduction:**

Knowledge of HIV status relies on accurate HIV testing, and is the first step towards access to HIV treatment and prevention programmes. Globally, HIV‐status unawareness represents a significant challenge for achieving zero new HIV infections and deaths. In order to enhance knowledge of HIV status, the World Health Organisation (WHO) recommends a testing strategy that includes the use of HIV‐specific antibody point‐of‐care tests (POCT). These POCTs do not detect acute HIV infection, the stage of disease when viral load is highest but HIV antibodies are undetectable. Complicating things further, in the presence of antiretroviral therapy (ART) for pre‐exposure prophylaxis (PrEP) or post‐exposure prophylaxis (PEP), other currently available testing technologies, such as viral load detection for diagnosis of acute HIV infection, may yield false‐negative results. In this scoping review, we evaluate the evidence and discuss alternative HIV testing algorithms that may mitigate diagnostic dilemmas in the setting of increased utilization of ART for immediate treatment and prevention of HIV infection.

**Discussion:**

Missed acute HIV infection prevents people living with HIV (PLHIV) from accessing early treatment, increases likelihood of onward transmission, and allows for inappropriate initiation or continuation of PrEP, which may result in HIV drug resistance. While immediate ART is recommended for all PLHIV, studies have shown that starting ART in the setting of acute HIV infection may result in a delayed or complete absence of development of HIV‐specific antibodies, posing a diagnostic challenge that is particularly pertinent to resource‐limited, high HIV burden settings where HIV‐antibody POCTs are standard of care. Similarly, ART used as PrEP or PEP may supress HIV RNA viral load, complicating current HIV testing algorithms in resource‐wealthy settings where viral detection is included. As rollout of PrEP continues, HIV testing algorithms may need to be modified.

**Conclusions:**

With increasing use of PrEP and ART in acute infection we anticipate diagnostic challenges using currently available HIV testing strategies. Research and surveillance are needed to determine the most appropriate assays and optimal testing algorithms that are accurate, affordable and sustainable.

## 1 Introduction

In the current era of immediate antiretroviral therapy (ART), and pre‐ or post‐exposure prophylaxis, confidently diagnosing HIV is becoming increasingly complex. Cases of diagnostic uncertainty can be confusing and distressing to both clinicians and patients, and can lead to difficult management decisions, particularly in regard to initiation of ART for treatment or prophylaxis.

ART has dramatically improved survival for people living with HIV (PLHIV) and globally there has been an improvement in treatment coverage 1. Viral suppression on ART confers an individual health benefit 2, 3, and a significant reduction in the risk of onward transmission 4, 5, 6, with an impact on HIV incidence at a population level 7, 8, 9, 10. In 2014, the Joint United Nations Programme on HIV and AIDS (UNAIDS) set the 90‐90‐90 targets, whereby 90% of people with HIV will know their status, 90% diagnosed will be on ART and 90% on ART will be virally supressed by 2020 11. By the end of 2017, globally 75% of PLHIV knew their status, but there were still 1.8 million new HIV infections 1.

Oral ART agents tenofovir disoproxil fumarate (TDF) and emtricitabine (FTC) as pre‐exposure prophylaxis (PrEP) for individuals at risk of HIV are highly effective at reducing HIV acquisition when taken correctly 12, 13, 14. In addition, the World Health Organisation (WHO) recommends one month of triple ART as post‐exposure prophylaxis (PEP) following HIV exposure 15. UNAIDS re‐set targets to reduce the global numbers of new HIV infections to less than 500,000 through ensuring that 90% of people at risk of HIV infection have access to comprehensive HIV prevention services by 2020, including PEP and PrEP 16.

Access to accurate HIV testing is crucial in order to direct PLHIV to treatment programmes and those who are HIV negative to appropriate prevention strategies. Tests to determine HIV status include those that measure HIV‐specific antibody and those that detect viral genetic material or proteins. Rapid HIV point‐of‐care tests (POCTs) are often used, particularly in resource‐limited settings, and also increasingly for self‐testing 17. These tests, which rely on the detection of HIV‐specific antibodies, do not diagnose acute HIV infection (AHI) 18. AHI is defined as the period prior to the development of detectable antibodies, and can be characterized by Fiebig staging 19, 20, 21, where in Fiebig I only HIV RNA is detectable, and in Fiebig II p24 antigen becomes detectable, a transient viral core protein. AHI plays a disproportionate role in HIV transmission events, and epidemiologic, phylogenetic and mathematical modelling studies suggest that AHI may be responsible for between 10% and 50% of all HIV transmissions 22, 23, 24, 25, 26, 27, varying by risk group and stage of the epidemic. Ideally, individuals at high risk or presenting with symptoms suggestive of AHI should have further diagnostic tests in addition to HIV‐antibody testing, such as nucleic acid amplification testing (NAAT) and/or HIV p24 *gag* viral core protein. However, most of these tests require venous blood sampling, sophisticated laboratory infrastructure and advanced personnel training, which are costly, time consuming and unavailable in many settings.

This scoping review was originally based on an invited symposium entitled “Strategies for diagnosing and managing AHI in the context of PrEP and immediate ART” at the 22nd International AIDS conference, July 2018. It has since been supplemented with evidence from clinical trials, observational studies, systematic reviews and international best practice guidelines, as well as updates from a similar session “HIV testing and management in the era of PrEP” at IAS 2019. In this scoping review, we aim to consider the difficulties in confirming HIV status using current testing strategies, and the reported challenges in confirming HIV status among people receiving PrEP or PEP, or those starting immediate ART in AHI using currently approved test kits and testing algorithms.

## 2 Discussion

### 2.1 HIV testing algorithms

WHO HIV testing guidelines recommend that specimens are first tested with the most sensitive rapid antibody POCT available. If this test is non‐reactive, individuals are considered HIV negative, whereas if this test is reactive, a second distinct assay is used 28. The United States (US) 29, European 30 and United Kingdom (UK) 31 guidelines all recommend an HIV diagnostic algorithm consisting of a laboratory‐based antigen/antibody (Ag/Ab) combination immunoassay followed by a confirmatory HIV‐1/HIV‐2 differentiation assay if positive. These guidelines also recognize that there are certain situations when a POCT may be recommended, such as settings where a rapid turnaround is desirable, or if venepuncture is unavailable or refused.

### 2.2 Diagnosis of AHI and immediate ART

Accurate HIV testing is necessary to allow timely identification of AHI and facilitate immediate ART initiation. However, identifying those with AHI is challenging, particularly since symptoms can be non‐specific or absent 32. Symptom and sexual behaviour risk scores have been validated in multiple settings across sub‐Saharan Africa to direct higher risk individuals to more intensive HIV testing with HIV RNA or p24 antigen 33, 34, 35. Targeted rather than non‐selective screening in this way gives the potential for substantial cost saving. Data from Lilongwe, Malawi have shown that rates of AHI were higher in symptomatic patients presenting to sexually transmitted infection (STI) clinics (1.0% of HIV‐seronegative patients) compared to HIV testing centres (0.3%)^36^. Among these patients, implementing a risk score‐based screening criteria would have identified 80% of patients with AHI by only screening 50% of the presenting patient population with HIV RNA, thereby conserving scarce screening resources 36.

Point‐of‐care Ag/Ab diagnostics that meet the WHO ASSURED (affordable, sensitive, specific, user‐friendly, rapid and robust, equipment free, delivered) criteria 37 are under development, but not widely available. These rapid tests use a lateral flow cassette to separately assay for both HIV antibodies and p24 antigen. Field studies of the US Food and Drug Administration (FDA)‐approved rapid Alere Determine™ HIV‐1/2 Ag/Ab Combo 38, 39, 40, 41, 42 have shown that antigen is rarely detected in whole blood specimens, with poor sensitivity for detection of AHI. The re‐formulated Alere^TM^ HIV Combo 43 has shown much improved sensitivity for detection of p24 antigen 44, 45, 46, 47, however, further evaluation of its use in clinical practice is required. Other options include diagnostic platforms for RNA testing, such as the Alere™ q HIV‐1/2 Detect 48 which has been validated for RNA detection among infants and children, the Cepheid GeneXpert^®^, the technology for which already has an existing presence in sub‐Saharan Africa where it is predominantly used for tuberculosis testing 49, and SAMBA 50 which is a dipstick‐based nucleic acid assay for the detection of HIV in whole blood developed for monitoring and diagnostic use in LMIC settings.

While there are clear benefits for all PLHIV to start ART irrespective of CD4 count, there are less randomized data on the urgency of starting ART among those with AHI. Early ART can increase the chance of CD4 count recovery 51, and there are also data that very early ART in AHI may limit viral reservoir seeding and confer enhanced likelihood of post‐treatment viral control of ART 52 although this remains rare. In the prospective RV254/Search010 study in Thailand, samples from clients with a non‐reactive Ag/Ab immunoassay were screened for AHI by pooled NAAT 53. Of the 112 participants with AHI (40% in Fiebig I), 111 initiated ART on the day of enrolment. The median time from HIV exposure to enrolment was 19 days 53. In this cohort there has been a trend towards participants treated in Fiebig I having a better clinical phenotype after two years on ART compared to later in acute infection or in chronic infection 54, 55. Despite encouraging observations in this cohort, interruption of ART among eight individuals followed for 24 weeks did not confer post‐treatment viral control 56.

The FRESH study in South Africa 57 follows initially HIV‐negative young women at very high risk of HIV acquisition with twice weekly HIV RNA testing. More than 1600 women have been enrolled, 71 of whom had incident HIV infection identified during the acute phase (> 70% in Fiebig I), with a median of four days since the last negative HIV RNA 58. Fifty‐seven of those with AHI initiated rapid ART within a median of one day from detection. A majority of those starting rapid ART (87%) did not develop detectable HIV‐specific antibodies on western blot, and nearly half (48%) of participants were consistently Western blot antibody negative up to 340 days of follow‐up 57. These data demonstrate that in the context of rapid ART initiation during Fiebig I, antibody tests for the confirmation of HIV infection are unreliable. Similar findings were reported in Thai participants initiating ART during AHI 59, 60, as well as among infants with perinatally acquired HIV receiving ART early in infection, where subsequent antibody testing remained negative at almost two years of age 61.

Understanding dissonant HIV test results in the setting of starting ART during AHI is a key community message to share with advocates and community leaders. In the era of rapid ART start there may need to be a new narrative around “testing positive” for HIV versus “being infected” or “living with” HIV. One patient from the RV254/Search010 cohort commented “although I have HIV, my blood test result is still normal just like a normal person… I still continue living my life as normal”^55^, ^62^. There is a complex dialogue about “normal” and being HIV negative, and management of negative HIV‐antibody test results for PLHIV can be difficult. The long‐term implications of remaining HIV seronegative maybe far reaching and if future ART interruption studies are explored amongst these cohorts there is a potential chance of seroconversion.

### 2.3 HIV diagnosis in the context of PrEP and PEP

It is important to rule out AHI prior to initiating PrEP, as starting dual ART with TDF/FTC in undiagnosed AHI may not control viraemia and carries the potential for emergence of drug‐resistance 63, 64, 65. In a meta‐analysis of 18 PrEP studies, six reported cases of TDF or FTC resistance, and the risk of developing resistance mutations was significantly higher in PrEP users who initiated PrEP during AHI 63. In another review exploring HIV resistance in PrEP studies, 3% of participants infected with HIV post enrolment had TDF or FTC resistance compared to 41% of those who had undetected AHI at enrolment 66. When the long‐acting injectable cabotegravir, which is currently in development for PrEP, was given to macaques in acute infection there were high levels of integrase resistance 67. These data highlight the importance of accurate testing to exclude AHI prior to initiation of PrEP. In some reported cases of HIV infection in the context of PrEP, resistance mutations may have been transmitted, that is, occurred secondary to exposure to resistant virus rather than selected for post transmission 68, 69.

International guidelines have recognized the need to adjust HIV testing algorithms in the context of PrEP. WHO guidelines state that it is critical to rule out HIV prior to starting PrEP and recommend a serial testing strategy which, in most LMIC, will involve a combination of POCTs. If the initial HIV serology test is negative without any clinical suggestion of AHI then PrEP can be initiated 70, and HIV testing is recommended every three months for the duration that the client remains on PrEP. If an HIV‐antibody test is inconclusive then a repeat is recommended 14 days later and discontinuation of PrEP should be considered. In the Centers for Disease Control (CDC) algorithm for determining HIV status for PrEP provision, a negative HIV‐antibody test (which may be an antibody POCT) with no signs or symptoms of AHI in the past four weeks means PrEP can be initiated 71. If there is concern about AHI then a laboratory Ag/Ab assay is preferred, with alternative options of HIV RNA or to retest for antibody in one month and defer the decision on whether PrEP can be started 71. The IAS‐USA guidelines recommend an Ag/Ab assay to determine PrEP eligibility, and if there is any suspicion of AHI then an HIV RNA assay should also be sent, and a repeat Ag/Ab test should be performed in one month 72.

Once PrEP has been initiated, frequent monitoring for HIV is important, with most guidelines recommending re‐testing at least every three months. This minimizes the risk of inappropriate dual therapy with TDF/FTC among PLHIV, and the subsequent risk of development of HIV drug resistance. However, it is unclear how well currently available HIV tests perform in the presence of PrEP. Case reports have detailed atypical HIV‐antibody test results in those taking PrEP 73, 74, 75, with the possibility of a delayed or reduced antibody response to HIV infection and suppressed viral replication. In the ADAPT trial (HIV Prevention Trials Network (HPTN) 067) that evaluated TDF/FTC PrEP in women in South Africa and men who have sex with men (MSM) in Thailand and USA, there were 12 new HIV infections 76. In 9 of the 12 cases, both of two POCTs were non‐reactive at the first HIV‐positive visit, including eight cases of AHI. Of these eight cases, five also had a negative laboratory Ag/Ab test. The viral load was ≤ 400 copies/mL in four of the eight AHI cases, but all were positive on a sensitive HIV RNA qualitative assay. In eight cases there was continuation of PrEP, including one case where PrEP was continued for three to four months post HIV infection; there were three cases of drug resistance to ART 76. In an assessment of HIV diagnostic tests in the ANRS‐IPERGAY study, the Abbott Architect^®^ Ag/Ab Combo test detected 85% of AHI but failed to detect two early infections, both which had HIV RNA of < 500 copies/mL 77. The BioRad Bioplex 2200 Ag/Ab assay missed three more infections, and the antibody POCTs performed poorly in AHI 77. In the Partners PrEP study, PrEP delayed the time to detect seroconversion but this delay was not associated with developing resistant virus 78. Eleven percent of seroconverters in the PrEP arm had an undetectable HIV RNA compared to 3% in the placebo arm 78. Overall, interpretation of HIV RNA and antibody test results can be more challenging in the context of PrEP, and confirming or ruling out a diagnosis can be difficult 79.

At the screening visit in HPTN PrEP studies, a combination of a POCT, instrumented Ag/Ab test, and an HIV RNA test are recommended for HIV diagnosis 80. At all follow‐up visits, one or two POCTs are performed as well as an instrumented Ag/Ab test. In any cases of discrepant results, confirmatory samples are taken and additionally tested for proviral HIV DNA from cell pellet samples. If needed, additional testing is performed at a third visit after a four‐week product hold to help determine HIV status 80. Presently, total HIV nucleic acid and proviral DNA testing are used only in research algorithms and are not feasible or available in routine care.

In large ongoing PrEP trials including the use of long‐acting injectables as PrEP (HPTN 083 81 and HPTN 084 82), broadly neutralizing antibodies 83 or vaccines 84, we would expect to see similar diagnostic challenges. These trials will help inform appropriate HIV testing algorithms pre‐ and post‐PrEP initiation, as well as preferred HIV diagnostic platforms and management decisions in cases of diagnostic uncertainty.

In situations where there are indeterminate results, Smith et al. have proposed three potential management options 85, 86. The first is to continue PrEP while confirming HIV status. This has the advantage of ongoing protection against HIV infection if negative, but would be inadequate therapy if HIV positive, with a risk of drug resistance. The second option is to discontinue PrEP to aid diagnosis with recommendation to use condoms, however, this may put HIV‐negative individuals at risk of acquiring HIV. The final option would be to intensify PrEP to triple therapy while performing additional tests to confirm status. If HIV positive, this would allow for the benefits of immediate ART, but would be unnecessarily exposing the individual to a third agent if HIV negative, and may make it more difficult to confirm or rule out HIV. Similar considerations on whether to continue or discontinue ART may be needed in those taking PEP with ambiguous HIV testing results.

HIV testing in the context of PrEP is particularly challenging in LMIC settings where there is limited access to advanced laboratory tests and infrastructure. In these settings sensitive and affordable rapid tests to detect acute infection are desperately needed to supplement current WHO guidelines, which only require an antibody POCT to rule out HIV 70. Additionally, relying on antibody tests to detect HIV during PrEP follow‐up is also likely to be inadequate. Cases of HIV drug resistance either occurring as a result of PrEP initiation in missed AHI, or due to the increasing rates of transmitted ART resistance in LMIC 87, could be particularly problematic in these settings as ART choice is likely to be limited, and alternative therapy options may not be available.

## 3 Conclusions

We can anticipate that with increasing use of PEP, PrEP and immediate ART, the currently available HIV diagnostic tests may be inadequate to reliably make clinical assessments and treatment decisions. Current routine rapid POCTs are unable to detect AHI, and some individuals starting ART in AHI may never have detectable antibody with these tests. There is no one easy solution to this, especially in resource‐poor settings where there is limited choice and availability of diagnostic tests, and the costs of implementing widespread additional HIV testing maybe unsustainable. Optimal testing algorithms to allow safe use of ART for prevention still need to be established, and future guidelines are likely to incorporate the use of sensitive rapid tests to detect AHI, including whole blood nucleic acid assays or rapid Ag/Ab tests. Information gained from ongoing large‐scale HIV prevention studies will provide insights into solutions for these diagnostic challenges.

## Competing interests

None: TE, EJS, MD, TN, MC, PP, GC, SER, JA, CB, SF.

## Authors' contributions

All authors contributed to the content and preparation of the manuscript, and approved the final draft. Specific contributions: TE drafted the manuscript, and performed a literature review; EJS, JA and CB provided expert opinion and revision of the manuscript for important content, MD provided expert opinion, specifically on WHO guidelines; TN and SER provided expert opinion, preparation and revision of the manuscript, specifically on immediate ART in AHI and experience in LMIC; MC provided expert opinion and revision of the manuscript, including in regards to HPTN study protocols; PP provided expert opinion and revision of the manuscript, specifically in regards to CDC guidelines; GC revised the manuscript with inclusion of patient perspectives; SF contributed to the preparation and revision of the manuscript, and provided expert opinion.

## Funding

No funding was received for the preparation of this manuscript.

## Disclaimer

The findings and conclusions in this report are those of the author(s) and do not necessarily represent the official position of the funding agencies. Use of trade names and commercial sources is for identification only and does not constitute endorsement by the Centers for Disease Control and Prevention.
